# Time Dependency of Psychotherapeutic Exchanges: The Contribution of the Theory of Dynamic Systems in Analyzing Process

**DOI:** 10.3389/fpsyg.2012.00253

**Published:** 2012-07-25

**Authors:** Sergio Salvatore, Wolfgang Tschacher

**Affiliations:** ^1^University of SalentoLecce, Italy; ^2^University of BernBern, Switzerland

**Keywords:** psychotherapy process, dynamic systems, non-linearity, self-organization, synergetics, pattern analysis, sequence analysis

## Abstract

This paper provides a general framework for the use of Theory of Dynamic Systems (TDS) in the field of psychotherapy research. Psychotherapy is inherently dynamic, namely a function of time. Consequently, the improvement of construct validity and clinical relevance of psychotherapy process research require the development of models of investigation allowing dynamic mappings of clinical exchange. Thus, TDS becomes a significant theoretical and methodological reference. The paper focuses two topics. First, the main concepts of TDS are briefly introduced together with a basic typology of approaches developed within this domain. Second, we propose a repertoire of investigation strategies that can be used to capture the dynamic nature of clinical exchange. In this way we intend to highlight the feasibility and utility of strategies of analysis informed by TDS.

## Introduction

Psychotherapy is a communicational flow unfolding irreversibly through time. In psychotherapy process, everything that happens occurs after and thanks to what has happened before, and paves the way for what follows. Psychotherapy is inherently dynamic, in essence it is a function of time – what happens in psychotherapy depends on time.

Insofar as one recognizes the time dependency of psychotherapy process, one has to conclude that the improvement of construct validity and clinical relevance of psychotherapy process research is intertwined with the development of models of investigation allowing dynamic mappings of clinical exchange. In this perspective, we cannot but agree with those scholars who, already more than two decades ago, called for the Theory of Dynamic Systems (TDS), conceiving of it as an indispensible source of theoretical, methodological, and technical concepts (Greenberg and Pinsof, 1986; Tschacher et al., 1992).

We do not believe that TDS has demiurgic power, that it could lead psychotherapy research beyond all its limitations; yet we think that TDS can provide a relevant contribution and address these current limitations. Therefore, we believe that process research has much to gain from a broader diffusion of TDS and its novel way of looking at clinical affairs.

This paper offers a contribution toward this goal. We focus on two topics. First, a brief introduction to main concepts of TDS is provided, together with a basic typology of approaches developed within this domain. The typology is not exhaustive, since it singles out the approaches of major interest for clinical research. For each dynamic model elaborated by the typology, we provide examples of clinical processes showing dynamic behavior. Second, we propose a repertoire of investigation strategies that can be used to grasp the dynamic nature inherent to clinical exchange. Each of these strategies is presented together with references to possible clinical applications.

Throughout the discussion of these topics, we aim at getting two complementary results, which we expect could promote clinical researchers’ commitment to TDS. On one hand, we intend to provide a systematic framework for the heterogeneous forms of dynamic analyses currently at stake. This should facilitate recognizing the conceptual and methodological utility of TDS for psychotherapy research. On the other hand, we intend to highlight the feasibility and accessibility of the strategies of analysis informed by TDS, as well as the heuristic valence of the results they are able to produce.

## What Every Clinician Knows

The time dependency of the clinical process is evident at different levels of observation. At the non-verbal level of communication, time dependency was highlighted by studies of the clinical relevance of the synchronization between patient’s and therapist’s body movements (Ramseyer and Tschacher, 2006) as well as between paralinguistic and semantic levels of speech (Tonti, 2007). Such “synchrony” is an instructive phenomenon because it shows how the clinical value of a given local pattern (a body movement, a speech tone, a latency in answering) depends on the temporal context in which it occurs.

Time dependency is evident at the semantic and pragmatic level of communication as well. The meaning of any event occurring within the therapist-patient communicational flow does not lie in the event, but in the connection between the event and the previous and following events. This general tenet is clearly shown by the way meaning is actualized within discourse (Linell, 2009). Take the following patient’s statement (*X*): “People have recently made me very upset.” Consider now that the *X′* previous statement was (1): “For a long time, I have accepted as normal that other persons were unkind with me. But now I have learned that I can and must claim respect” or (2) “I need help. I get angry too easily – everything ticks me off.” Obviously, the sequences 1-*X* and 2-*X* produce two different meanings. In the former case, the patient is speaking of a positive, ego-syntonic request, in the latter of a problem motivating her to ask for being helped. In the former case we attribute the emotion of anger as a marker of the capacity of self-esteem; in the latter the same emotion acquires the value of a marker of a low capability of impulse control.

At a more macroscopic level (almost) any clinician would agree with the statement that the relationship between therapist and patient is not constant over time. Rather, it is a field with its own historical development, which affects actions and reactions of the therapeutic dyad in ever-changing ways. Take a therapist’s intervention Y (e.g., the reference to the patient’s inner state; a self disclosure; an interpretation): Y may not have the same clinical impact regardless of the moment in therapy when it is performed. Rather, its clinical valence varies (quantitatively as well as qualitatively) in accordance to the history of the relationship between therapist and patient. For instance, the identical interpretation provided at the first session could be understood as an intrusion by the patient, whereas later as a gift.

A further aspect to be mentioned concerns the trajectory of change. For clinicians it is obvious that the development of the therapeutic situation is all but constant. Clinical work often proceeds in a wave-like fashion, with moments of acceleration alternating with moments of stall. Symptoms trend to decrease fast in the initial part of therapy (Lambert and Ogles, 2004); in some cases they may reappear in critical phases. Relevant changes suddenly arise, whereas other periods of clinical work are seemingly unproductive (Laurenceau et al., 2007).

### Dynamic analysis – a long-standing claim

Being aware of facets such as the ones mentioned above, several researchers have become dissatisfied with the linear, time-independent models of clinical investigation. This is not only a recent topic. Almost a quarter century ago, Stiles et al. (1989) formulated a radical criticism of the traditional paradigm of process research, which aligned psychotherapy process with pharmacotherapy. The authors named this paradigm “drug metaphor”:

An investigative paradigm … [which] views psychotherapy as comprising active ingredients, supplied by the therapist to the client, along with a variety of fillers and scene-setting features. The supposed “active ingredients” are process components – therapeutic techniques such as interpretation, confrontation, reflection, self disclosure, challenging assumptions, focusing on affect, effort to give support, or (more abstractly) empathy, warmth, or genuineness. If a component is an active ingredient, then a high level of it is supposed to yield a positive outcome. If it does not, the ingredient is presumed to be inert. (p. 37)

The drug metaphor is a clear example of a linear, molecular, additive conception of process. According to this conception (a) process and outcome are distinguishable, with the former working as cause of the latter; (b) process ingredients are known, substantive elements, isolable as discrete contents of the process, implemented in accordance with independent technical procedures and having always the same effects on the patient throughout the process (Stiles et al., 1989; see also Stiles and Shapiro, 1994).

These assumptions are clearly an oversimplified model of psychotherapy, overlooking the contextual, holistic, contingent, non-linear, and circular nature of clinical settings (Shapiro et al., 1989). Clinical exchange is affected by a large number of factors, far more than the ones psychotherapy research was able to isolate (*contextuality*; cfr. Bickhard, 2009). Above that, what is relevant are not the elements as such, but their interaction, i.e., their working as part of a whole (holism; Slife, 2004; Valsiner, 2007; Salvatore and Valsiner, 2010a). Consequently, no element can be thought to possess an invariant clinical valence throughout the process. Rather, its impact on the process is mediated by the field – namely, the set of co-occurring elements (*non-linearity*; Barkham et al., 1993). Moreover, the idea of technical ingredients implemented within the process, but independently of the process, contrasts with the obvious clinical observation that the patient is not merely the terminal of the therapist’s action, but circularly is also the trigger of the therapist’s action (*contingency*; see for example Gonçalves et al., 2010). Finally, as the debate on the therapeutic alliance has highlighted (Colli and Lingiardi, 2009; Horvath, 2011), the unidirectionality of the linkage between process and outcome is not tenable: process and outcome are circularly linked, with the former causing as well as being caused by the latter (*circularity*; Greenberg and Pinsof, 1986).

In terms of principles, the acknowledgment that linear assumptions are clinically inconsistent does not mean that analyses based on them must be considered invalid by definition; rather, the validity of linear analyses has to be constrained to the conditions of observations within which the assumptions of linearity can be tenable (Salvatore and Valsiner, 2010a). This is true in classical physics, where linear models can be trusted insofar as they are constrained within certain conditions of observation. In the case of psychotherapy research, the identification of such conditions is a matter of discussion in the domain of outcome studies (Westen et al., 2004); yet it is hard to maintain in the case of process research, in particular the process research being aimed at modeling mechanisms of change (Tschacher et al., 2012). This has raised the demand for dynamic analyses, i.e., investigations more consistent with the time dependent nature of clinical process. Consequently, this has entailed turning to the TDS as the best candidate to address such demands.

Clinical researchers have adopted a variety of models present within the TDS domain. Barkham et al. (1993; see also Stiles et al., 1992) focused on the temporal variation of the rates of change of targeted clinical parameters (in their study, the intensity of 10 personal problems, monitored by the patients). Such second order change was modeled in terms of a quadratic (i.e., non-linear) function showing how the velocity of change varied throughout therapy.

Greenberg and Pinsof (1986) referred to chaos theory.

Chaos theory’s image of patterned complexity offers a far better picture theory (Hansen, 1958) to guide our research efforts than does experimental design’s billiard ball determinism image of direct and linear causality. An alternative to experimental studies in psychotherapy is a research approach which recognizes the complexity of the psychotherapeutic process and attempts to analyze the complex unfolding of moment by moment performance of people in specific states and contexts. (p. 8)

In the same period, Schiepek and Tschacher (1992) proposed synergetics (Haken, 1992) for modeling the non-linearity and self-organization of clinical processes.

Each of the previous examples shows that the non-linear processes and phenomena of self-organization occur everywhere within the traditional areas of the research and practice of clinical psychology (…). In order to gain an understanding of the dynamic of evolution of such systems, theories of non-linear systems and especially synergetic conceptualizations will be necessary in the future (…). It should be clear by now that the synergetic approach to phenomena treated by clinical psychology neither leads to physicalist reductionism nor means mere metaphorical thinking. (p. 15)

### The current scenario: Unfulfilled promises?

These pioneering references to TDS paved the way to further studies. Schiepek and colleagues (Kowalik et al., 1997; Schiepek et al., 1997) analyzed the temporal series of indexes mapping a singular therapeutic dyad, and arrived at the conclusion that the description of the psychotherapy process produced by their measures suggested the characteristics of chaotic dynamics – namely, sensitivity to initial conditions and presence of strange attractors. Tschacher et al. (2000) applied a time series analysis of session-wise clinical indexes in 91 therapeutic dyads. In so doing, they elaborated prototypic patterns of change that differentiated the dyads in a clinically meaningful way. From within the theoretical frame provided by synergetics, Tschacher et al. (1998; see also Tschacher et al., 2007) highlighted the progressive synchronization of therapists’ and patients’ non-verbal behavior and how this trend is associated with the clinical outcome of the therapies. Recently, Nitti et al. (2010) analyzed how meanings combine with each other moment by moment in the communicational flows between patient and therapist. The indexes obtained by this kind of sequential analysis allowed to discriminate clinically good from unfavorable sessions as judged by an external independent criterion (100% success in classification).

The mentioned studies represent different – to some extent heterogeneous – approaches to the dynamic analysis of psychotherapy process. Taking them as a whole, however, they highlight the heuristic potential of clinical research informed by TDS. Nevertheless, one must acknowledge that TDS has had scarce impact on psychotherapy research and has remained a niche phenomenon. To date, it has not produced the large-scale conceptual and methodological innovations it has set out to achieve. To give a few examples of this situation, consider the methodological chapter (Hill and Lambert, 2004) of the recent edition of Bergin and Lambert’s *Handbook of Psychotherapy and Behavior Change*, which does not make any explicit reference to TDS and is limited to mentioning pattern and sequence analysis. Five years later, the Special Issue of *Psychotherapy Research* devoted to methodology shows a similar picture, with just 2 of 24 articles (one of which written by one of the authors of this paper) addressing the issue of time dependency of psychotherapy.

The missed opportunity to integrate dynamic approaches in clinical research may have various causes. Laurenceau et al. (2007) singled out two aspects. Firstly, analyses informed by TDS require a level of methodological and technical expertise that does not usually belong to the clinical researcher’s repertoire of competences. Secondly, dynamic analysis often requires large datasets frequently unavailable in psychotherapy process research. We agree with the authors that these two aspects may play a role, yet would not consider them as the main problems. Comparing current studies with those 20 years ago, one can easily observe an impressive development of methodological and technical sophistication – and the same may be said for the quality and extension of datasets. Some of these developments are at least as complicated and specialized as the ones entailed by dynamic analysis. Therefore, one would rather conclude that the availability of methodological and technical resources is not a constraint for the commitment to dynamic analysis.

This suggests that there are other causes for the missed development of dynamic analysis in the field of psychotherapy research. In particular, we singled out two factors. First, we believe that the shift of paradigm entailed by dynamic analysis is a relevant obstacle: The dynamic approach is grounded in a more general formal logic of analysis using conceptual objects such as phase space, attractors, probability of transitions, and the like. These concepts, in being not domain-specific, are quite far from clinical experience. Sciences like physics have shown that this level of abstraction is a way of empowering knowledge. Nevertheless, the idea of clinical knowledge as produced by abstract, formal models stands in sharp contrast with the empiricist paradigm of mainstream clinical research that tends to attribute meaning to constructs only insofar as they are linkable with, or derivable from, observables (Salvatore, 2011). Second, a state of fragmentation characterizes dynamic analyses of psychotherapy process. As mentioned, TDS is not a homogeneous model; rather, it is a family of different approaches, reflecting standpoints and applicative interests of various scientific domains (e.g., physics, chemistry, biology, neurophysiology, meteorology). The introduction of TDS in psychotherapy process research has mirrored this pluralism. Clinical researchers have been making references to a multiplicity of concepts (e.g., non-linearity, non-stationary equilibrium, bifurcations, emergence, strange attractor, dimensionality of the phase space, control parameter, synchronization, fractals, and so forth), representing as many different ways of analyzing clinical exchange in the light of the TDS’ general tenet of time dependency. Such variety does not necessarily equate to richness. So far, the various applications of TDS in psychotherapy research have followed parallel trajectories, unfolding only inside a few niches of scarce reciprocal communication. A reflection on the relationship among these applications is not yet available.

The application of concepts derived from chaos theory (Tschacher et al., 1997) and from the theory of autopoietic systems (Maturana and Varela, 1980) are examples for this: both applications have shown interesting results in the clinical field of systemic therapy (Kowalik et al., 1997; Schiepek et al., 1997); yet, the issues of to what extent, in clinical research, they are complementary, integrable, or competitive approaches is not deepened. The lack of this kind of reflection does not prevent developing specific programs of research. Yet it hinders pointing out the heuristic potential of dynamic analysis of psychotherapy process, hence the possibility that TDS could be targeted by clinical researchers with a more widespread and systematic commitment.

## Dynamic Models

### General definition

The defining characteristic qualifying a phenomenon as dynamic is dependence on time. This means: a phenomenon is dynamic if, in order to understand it, one has to take the temporal dimension into account. To clarify of a possible point of misunderstanding: It is obvious that any phenomenon, by definition, unfolds in time. Yet, not all phenomena are dynamic, because not all phenomena require reference to their temporality in order to have their functioning understood. Obviously, the movement of a lever unfolds through time, yet we need not refer to time for describing (and foreseeing) the extent of the force we have to implement in order to raise a certain weight. Therefore, the movement of a lever is not a dynamic phenomenon. Many clinical models treat their objects as non-dynamic phenomena. For instance, the interpretation of personality disorders in terms of dysfunction of meta-regulative abilities (Dimaggio and Semerari, 2004) is an explanation not entailing a reference to time.

This consideration allows to specify a further point. To be precise, not the phenomenon *per se* is dynamic or not, but the *model of it*. This is to say that one can model a phenomenon both dynamically or not dynamically. The point is which kind of model – dynamic or non-dynamic – provides a more efficacious and efficient representation of the behavior of the phenomenon. In the case of a lever, the non-dynamic model is efficacious enough to disregard the reference to time. Our basic thesis is that this is not the case when psychotherapy process is at stake.

### Theory of dynamic systems

Theory of Dynamic Systems is a network of mathematical concepts aimed at modeling phenomena once their temporal dependency is recognized. The dynamic approach, however, does not coincide with TDS: one can conceive of a phenomenon as dynamic without using TDS. To the contrary, the adoption of TDS entails the previous assumption of the dynamicity of the phenomenon. When this assumption can be made, the phenomenon may be modeled by TDS.

A dynamic system is a mathematical model of the phenomenon. The phenomenon is defined as the response to an external input, which depends both on the input itself and on the inner state of the system. In its more general form, the law that maps the evolution of the dynamic system is given by the following system of differential equations:

$$\begin{array}{rc} X\left(t\right) & =\left(x_{1}\left(t\right),\;x_{2}\left(t\right),\ldots ,x_{n}\left(t\right),t\right)\\ dX\left(t\right)/dt & =f\left(x\left(t\right),x_{0},u\left(t\right),t\right) \end{array}$$

where *X*(*t*) is the vector of the state variables *x*_1_(*t*), *x*_2_(*t*), …, *x_n_* (*t*); *dX*(*t*)/*dt* is its variation with respect to time, *x*_0_ provides its initial conditions, and vector *u*(*t*) is the input of the system at time *t*. Thus, the first equation expresses the state of the system as a vector of a certain number of state variables, each of them depending on time. The second equation maps the variation of the state variables as a function *f* of the state of the system at time *t*, of the input at time *t* and of the initial conditions.

In sum, the system of equations describes the dynamic system as a function of the temporal evolution of its inner state and input, both depending on time. This function, once assigned to the state of the system at an initial moment *t*_0_, allows to determine the state of the system at any subsequent instant of time. This is to say that the mathematical function is the formal law of the evolution of the phenomenon.

When the system’s behavior can be mapped in terms of sequences of discrete states, in its simplest version the model assumes the form of the following equation:

$$X_{t+1}=r\;X_{t}$$

expressing how the state of the system at a given moment of time *X_t + 1_* depends, by a function *r*, on the state of the system at the previous moment of time *X_t_*.

For the sake of the following discussion, it is worth adding that the mathematical description of the evolution of the system can be depicted in terms of geometrical representation, by drawing the system’s evolutive trajectory in phase space. This is a “shaped” space spanned by the degrees of freedom of the system as dimensions (i.e., coordinate axes): the points of phase space comprise all possible states of the system.

### A typology of dynamics models

There are different models of dynamic systems. Following Lauro-Grotto et al. (2009), here we focus on three types.

#### Periodicity

Periodic behavior is the first type of dynamic system of interest to our discussion. The perpetual motion of a pendulum (in the frictionless case) is the classical instance of this kind of dynamics. The position and velocity of the pendulum change with time, therefore it is a dynamic system. In addition, this change follows a cycle that remains constant at a larger temporal scale. The time evolution of a periodic system can be represented by a closed orbit in phase space, with every point representing the position of the system at a given instant. Therefore, the system changes positions at every instant, and returns to identical positions after a certain period.

This characteristic of the periodic behavior makes it a linear and stationary system, i.e., a system whose dependence on time is stable, does not change as the system evolves. At a formal level, the linearity is expressed by the fact that the temporal evolution function describing the state of the system is defined by a first order equation(s) (i.e., equation having the form of polynomial of first order – e.g., *y* = *ax* + *b*). Incidentally, the linear dynamic system preserves the property of compositionality, that is the fact that its evolution can be described step by step, taking the state reached at a given time point as starting point for the subsequent computation.

The interesting property of the periodic trajectory is that it shows a kind of behavior that appears to change with time – i.e., the velocity of the pendulum changes instant by instant, – yet is globally stable, in the sense that the system will return to the same point of (unstable) equilibrium.

Periodicity characterizes many aspects of living systems (e.g., the sleep-wake cycle, the metabolism, the seasonal trends of many biological parameters). Examples of periodic behavior are known in the clinical realm as well – the bipolar syndrome may be taken as prototypical; another example is given by the alternation of moments of grandiosity and moments of low self-esteem, feeling of incompetence and fragility characterizing some forms of narcissistic personality disorder (Dimaggio and Semerari, 2004). Periodicity is also found in psychotherapy process research, e.g., therapeutic alliance. The initial assumption of a linear linkage between this construct and clinical effectiveness of psychotherapy has been currently overcome by the more sophisticated vision that views the therapeutic alliance as a cyclic process of ruptures and repairing (Safran and Muran, 2000). According to this vision, what is clinically significant is not the absolute trend of the alliance, but the capacity of the therapeutic dyad to systematically cooperate in order to repair the inevitable micro-ruptures of their bond – that is to say the capacity of the therapeutic dyad to produce a virtuous cycle of separation and closeness. Accordingly, therapeutic alliance is better understood and studied in its clinical impact insofar as it is mapped as a periodic dynamics, which alternates between positive and negative poles.

The Therapeutic Cycles Model (TCM, Mergenthaler, 1996; Lepper and Mergenthaler, 2005, 2007; Kraemer et al., 2007) is a well-known conceptualization of psychotherapy that explicitly conceives the clinical process in periodic terms. According to TCM, psychotherapeutic work alternates between phases of emotional activation (emotional tone) and phases of cognitive elaboration (abstraction), whose combination across time draws a cyclical trend, articulated in four recursive steps (in the terms of the model: relaxing, experiencing, reflecting, connecting).

Bucci’s (1998) Referential Activity Theory (RAT) is a further model of psychotherapy process explicitly referring to a periodic dynamics. Referential activity is the function of the mind enabling the subject to mentalize the sub-symbolic components of emotional activation, by bringing them into language. Referential function, then, reflects an ever-changing dialectic between the sub-symbolic emotional activation (primary process, in psychoanalytic terms) and symbolic elaboration (secondary process, in psychoanalytic terms). Bucci (1998) has shown that clinically significant sessions of psychotherapy process present a cyclic pattern with phases of high referential activity (when secondary process is preeminent) alternating with phases characterized by low referential activity (when primary process is preeminent). This pattern is consistent with the author’s psychoanalytically informed theory assuming the mind oscillates between phases of affective activation and phases in which affect is elaborated by reflective thought.

In the light of the above considerations, three general observations can be made. Firstly, one may conclude that modeling psychotherapy process as periodic dynamics reflects a trans-theoretical standpoint. As a matter of fact, TCM and RAT are grounded in different clinical theories – cognitive theory, the former; psychoanalysis, the latter. Secondly, it is worth noting that both TCM and RAT are normative models of process rather than mere methods of analysis. In other words, both TCM and RAT adopt periodicity as a way to define the ideal model of psychotherapy. And both of them analyze concrete sessions in terms of their distance from an ideal-normative model. If one wishes to generalize this aspect, one may conclude that TDS provides not only procedures of analysis, but also conceptual tools for theoretically modeling the clinical phenomena. Finally, TCM and RAT jointly highlight that the clinically relevant dimensions, i.e., how clinicians and researchers represent and understand psychotherapy, may have to be conceived of as second order phenomena, products of non-linear combinations of more basic mechanisms. TCM identifies these mechanisms in the emotional tone and in abstraction; RAT refers to the psychoanalytic constructs of primary and secondary processes. These mechanisms combine their effects systematically, throughout the course of psychotherapy. Yet the result of these combinations changes over time, thereby producing different patterns, each of which mirrors a certain reciprocal equilibrium of the components. This way of conceiving of clinical process has a significance that goes beyond TCM and RAT. According to this standpoint, periodic models might help to understand various aspects of clinical process – in particular, those aspects that one can interpret as high-order phenomena reflecting the cyclic combination of lower-order mechanisms (e.g., defense mechanisms, attitudes toward the clinical situation, and the like).

#### Non-linearity

Non-linearity is a term used with several meanings. In this context, we consider it in accordance with its basic significance: a system is non-linear if the ratio between a certain variation of the independent variable, Δ*X*, and the corresponding variation of the dependent variable, Δ*Y*, is not a constant. Non-linearity is not confined to dynamic systems; yet it is a characteristic qualifying a relevant class of such systems. Non-linear dynamic systems are given by polynomial equations of order higher than one (such as quadratic or cubic exponential equations).

Despite the fact that clinicians usually tend to consider and empirically investigate clinical change as a linear and incremental process, there are many clinical and empirical indications casting doubt on such a traditional attitude. Hayes et al. (2007) listed some of these issues. Various studies have documented that, following dramatic and traumatic events, the life trajectory does not evolve in coherence with the local effect of the trauma, i.e., in terms of the onset of pathological conditions (so called Post-traumatic Stress Disorders). In various cases, people are shown to be able to regain their pre-traumatic condition. In further cases the traumatic event is shown to be the premise and the means for reaching an even better psychological condition (the so called Post-Traumatic Growth). Other studies have shown that people with clinical problems – e.g., with problems of substance abuse – can fundamentally change their condition as the result of a sudden, rapid, and global transition. Moreover, such changes are often preceded by periods of worsening of the clinical condition. Other clinicians have highlighted how the clinical improvement can follow a threshold mechanism, as a consequence of the accumulation of a set of eliciting conditions, yet none of them sufficient to be singularly effective. Finally, many studies on psychotherapy process-outcome show that clinical improvement – often measured in terms of the level of symptomatology – does not evolve homogeneously throughout the course of the clinical treatment. Rather, at least in good-outcome cases, most of the improvement occurs in an early phase, followed by lower rates later on (Lambert and Ogles, 2004).

Several studies of psychotherapy process have shown that clinical parameters can follow regular, but not linear trends. We have already referred to an analysis representative of this kind: Barkham et al. (1993). These authors assumed, as appears reasonable from a clinician’s perspective, that progress in psychotherapy is never constant, passing through sudden accelerations followed by moments of stasis. Consequently, they hypothesized that the trend of patients’ relevant clinical problems (as assessed by patients themselves) in the course of psychotherapy followed a *U*-shaped trajectory, best modeled by a quadratic function. Their findings supported their hypothesis, even if they underline that other non-linear curves (cubic or of higher order) may be equally appropriate.

It is worth noting here that this kind of study highlights how clinically meaningful parameters of psychotherapy process may not show linear trajectories as would be assumed by the drug metaphor paradigm; they still may follow a regular trend, yet are depictable only if mapped as a non-linear model.

**Figure 1:**
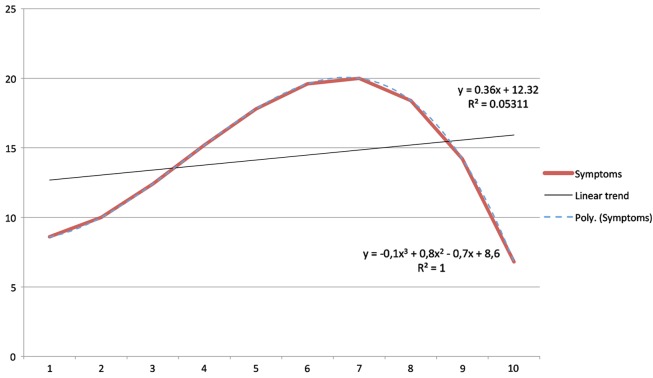
**An example of non-linear trend**.

The description of trends as non-linear trajectories can present some difficulties. The majority of statistical tools that clinical researchers have at their disposition are based on the assumption of linear relationships among variables. Therefore, questioning this assumption casts doubt on the use of these analytic tools. Yet, such costs are associated with a relevant advantage, given that the application of non-linear models may highlight clinically relevant regularities not accessible by the use of first order models. An ideal example of this situation is illustrated by Figure 1, which shows a hypothetical relationship between two parameters (say, time, and symptoms). Assuming a linear model, the parameters appear unassociated. If one assumes, however, that the two measures have a non-linear relationship (namely, a cubic relationship), then the association becomes apparent in all its force.

#### Deterministic chaos

Chaos appears in non-linear dynamics with specific values of control parameters in the set of equations defining the system. As consequence of these values, the system enters a condition of apparent randomness. Chaos can therefore be understood as this specific form of erratic, disordered behavior, resulting from deterministic rules – the ones defined by the set of equations. Chaos theory is aimed at representing the apparent disorder by showing its inner regularity.

Take the following equation

$$x_{t+1}=r{x_{t}}^{*}\left(1-x_{t}\right),\text{with}\;0<x<1\;\text{and}\;0<r<4$$

It can be used for mapping the temporal evolution of a population (where *x* = 0 means extinction and *x* = 1 means the maximum possible population) in a given environment characterized by given values of the death rate, reproduction rate, and starving rate (jointly expressed by control parameter *r*).

**Figure 2:**
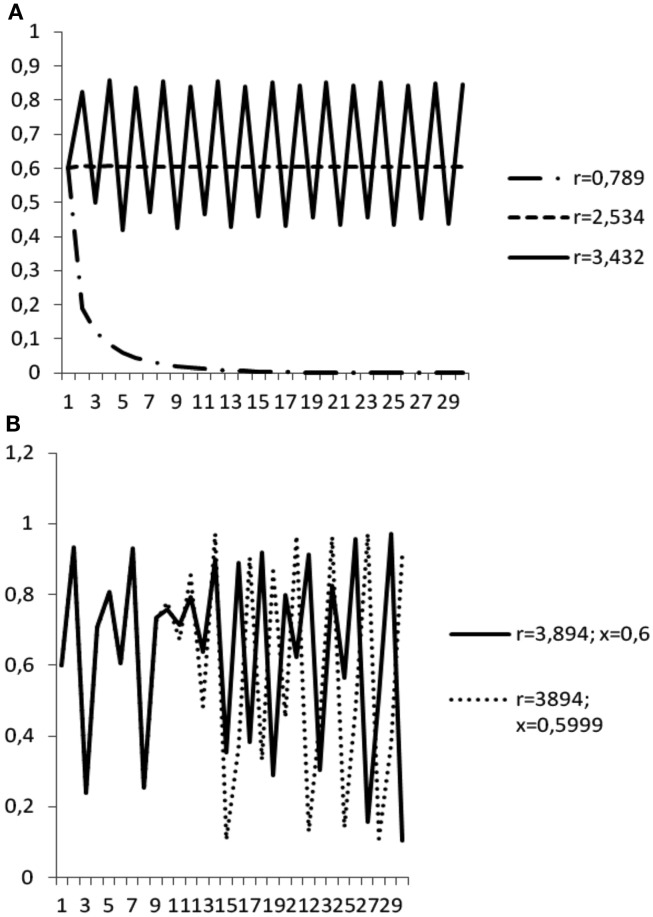
**The bifurcation route to chaos**. **(A)** The dynamics of the system in a function of the control parameter (*r*). A bifurcation emerges when *r* > 3.45 (approximately). **(B)** The butterfly effect: the two systems are regulated by the same equation, have the same control parameter (*r* = 3.894 – beyond the threshold for chaotic dynamics: *r* > 3.45) and very similar-but not identical-starting points (*x* = 0.6; *x* = 0.5999).

Regardless of the starting value of *x_t_*, if *r* < 1, the system evolves toward the value *x* = 0, which therefore represents the fixed point of stable equilibrium (i.e., the population will eventually be extinct). For 1 < *r* < 3, a stable fixed point with *x* ≠ 0 emerges – more precisely the fixed point is given by the value: (*r* − 1)/*r*. For 3 < *r* < 3.45 (approximately) the system undergoes a so called *bifurcation*: instead of a single fixed point it expresses two different fixed points, with an oscillation of the population between the two values (Figure 2A). As *r* further increases, more and more bifurcations take place – thus, the oscillation’s period increases to 4 values, then 8, 16, and so on. Yet, when *r* goes beyond 3.57 (approximately) the system enters the chaotic regime: from almost all initial values of *x*, there is no finite cycle of oscillation – the system shows an erratic, apparently random behavior (Figure 2B). This is a classical way in which a transition from a non-chaotic to a chaotic regime can take place, known as the *bifurcation route to chaos*.

Chaotic dynamics has two main properties. The first is *sensitivity to initial conditions*. According to this property, even a very small change in initial condition may create large consequences over time, like the metaphor of a butterfly’s wings in Cape Town causing – after a while – a tornado in New York (therefore, the sensitivity property became popular as “butterfly effect”). Figure 2B reports the trajectory of two systems regulated by the same equation, having the same control parameter (*r* = 3.894; note: beyond the threshold for chaotic dynamics) and very similar – but not identical – starting points (*x* = 0.6 and *x* = 0.5999, respectively). As one can see, after *t* = 9, the two trajectories diverge – and after *t* = 15 the difference among them becomes dramatic. This characteristic of chaotic dynamics is responsible for the intrinsic unpredictability of the system after a long enough period. Even tiny differences in the measurement of the initial condition – and no measure can be absolutely precise – will lead to a marked divergence of the trajectories.

A second property of chaotic dynamics is the *density of the periodic orbit*. The trajectory of a chaotic system stays inside a circumscribed portion of the phase space (i.e., strange attractor). This means that the system does not assume all possible values represented by the infinite number of points of phase space; to the contrary, it reduces its variability in the course of time. Nevertheless, the chaotic system is not periodic: the system will never present the same state twice. In geometrical terms, the system never passes through the same point of phase space twice. This means that however small is the sub-region of phase space to which the orbit of the chaotic system is confined, one will find infinitely many points, each of these representing the state of the system in a generic instant *t*. The presence of strange attractors leads to the recognition that even if the chaotic behavior seems random, this actually is the expression of a different, more complicated order. A chaotic trajectory shows a *quasi-periodic* course: it reproduces similar cyclic behavior over the time, yet always different to a certain extent. Therefore, a chaotic system can be predicted, if one assumes a conception of prediction aimed at identifying the region of the phase space the dynamics constraints itself within a temporal range, rather than its point-like position in a given instant *t*.

Chaos theory is a popular notion and a source of fashionable metaphors. Its major worth lies in the possibility to recognize how phenomena that show a disordered, erratic behavior at the descriptive level, may yet turn out to be the product of deterministic rules – i.e., a more sophisticated form of order, which may then be addressed. Technical and theoretical reasons, however, advise to be cautious on the possibility of adopting it as a model of psychotherapy process. From a technical point of view, chaos analysis requires very large datasets, with many observations at high frequencies of highly precise measurement. In fact, only under such conditions quasi-periodic trajectories become visible. From a theoretical point of view, modeling a phenomenon in terms of chaotic dynamics assumes the possibility of varying externally the value of the parameter (the defined control parameter) the transition to chaos depends on. In our previous example, *r* is the control parameter, whose fine manipulation shifts the system from a periodic to a chaotic regime. Yet, the assumption of an external, fine-manipulable control parameter is hardly tenable in the case of psychotherapy process. Which should be this parameter? Above all, in which sense could it be considered external? Interestingly enough, the authors of what is in our knowledge the only example of application of chaos theory to psychotherapy, arrive at recognizing this point as an unsolved issue of their study.

The sudden chaoticity jumps we observed are not transitions in the sense of real “phase transitions,” as this would require a change of at least one control parameter. In order to observe such changes, experimental manipulation of the control parameters would be necessary, which has not been realized. In general, the analogy between experimental and therapeutic process is applicable to a very limited extent, because the important sources for change during therapy arise from the client and the client-therapist relationship and not from outside. The therapist is part of the therapeutic system and not an externally controlling source of an independent variable. Theoretically it is not yet clear what might be a suitable control parameter for therapeutic phase transitions. A possible candidate might be the client’s motivation for change, though this is not an environmental parameter like the energy input for the laser or the temperature gradient (…) but a parameter inherent in the process (Kowalik et al., 1997, p. 212).

A possible route out of the impasse of *verifying* chaotic dynamics is trying to falsify all plausible non-chaotic models of a given empirical time series. This can be done by statistical tests using surrogate versions of the empirical time series one wishes to model, a method similar to Montecarlo analysis (Ramseyer and Tschacher, 2010). Tschacher et al. (1997) have performed such an approach with 14 exceptionally long time series of psychotic symptoms. They found that in 8 of the 14 documented time series of psychotic patients, the hypothesis of a complicated non-linear, possibly chaotic dynamics underlying the temporal evolution could *not* be falsified (cf. Pezard et al., 1996).

Finally, there is a more general theoretical issue to be considered in the case of chaotic dynamics. Chaos theory is aimed at recognizing the unitary deterministic source of phenomenically erratic dynamics. Yet in the clinical realm the inherently high variability of clinical phenomena is not merely conceivable as mirroring unitary erratic dynamics. Rather, it is more convincingly interpreted as due to the huge number of dimensions that generally interact in complex human affairs.

#### Self-organization

Similar to the chaotic system, the self-organization model concerns order arising from a non-linear system. Yet, differently from the chaotic system, a self-organized system is characterized by a huge amount of interacting microelements – e.g., the neurons in a neural network – that can behave either in a deterministic or in a stochastic (noisy) way.

Self-organizing systems are capable of creating forms of persistent order spontaneously, “by themselves.” The self-organization phenomenon often arises as a sudden reduction of the variability of the behavior of the systems’ elements. Their microscopic pattern formation produces the emergence of structures of order (i.e., organization) at the macroscopic, phenomenal level. Thus, reduction of variability and emergence are the two main markers of self-organization. The elements of the system, which until a critical moment have acted independently of each other, suddenly start to act as if in a linkage of close reciprocal dependence. In this sense the order emerges from within the system, rather than being imported from outside – and consists in the structure of reciprocal dependence among the elements of the system.

In clinical psychology, several authors have made reference to synergetics (Haken, 1992) as a theoretical model enabling to model phenomena of self-organization. Synergetics focuses on complex systems, i.e., those constituted by a very high number of microscopic components functioning in a stochastic way (e.g., the molecules of a fluid). Under specific conditions – described by values of a control parameter – the behavior of the micro-components starts to follow a common rule: an *order parameter* has emerged which now governs the dynamics of the system. According to the terminology of the theory, the micro-components have been “enslaved” by the order parameter. In so doing, a coherent pattern emerges as a global property characterizing the system as a whole. The order parameter thus represents a global variable that comprehensively describes the system’s dynamic behavior. In the physical domain, a prototypical example of a dynamic system exhibiting an order parameter is the laser: above a critical value of the control parameter, the photons exit stochastic behavior and become enslaved to a common rule that transforms stochasticity into a single mechanism with specific properties of order.

Ramseyer and Tschacher (2011; see also Tschacher et al., 1998) have adopted synergetics as a framework for analyzing non-verbal communication between patient and therapist in psychotherapy. They assume that the patient-therapist dyad’s non-verbal interaction works as a self-organizing system characterized by the emergence of synchronization of the two participants’ movements. This synchrony is viewed as the macroscopic marker that reflects the enslaving of body micro-movements by an order parameter. Authors underline the clinical relevance of synchrony, conceived of as a clinical marker of the efficacious working of the therapeutic relationship. They consequently report significant associations between synchrony and favorable outcome of the respective psychotherapies.

A similar approach has been adopted by Salvatore et al. (2006/2009) in their analysis of the verbal interaction between therapist and patient of a 123-session good-outcome psychotherapy. The study assumes that sensemaking – and therefore sensemaking within psychotherapy – works as a *self-organizing system* because it emerges *from within* the dialogical dynamics, as a product of the discourse’s own functioning, rather than as a consequence of an external intervention (i.e., as a consequence of “meta-stipulation” among the actors in the discourse about a set of assertions, fixing the semiotic ground of the relationship). Meaning can be seen as the constraints that the communication imposes on the virtually infinite possibilities of combinations of signs. According to this general assumption, at the initial time (*t*_0_) the communication between therapist and patient can be considered a system with a maximum extent of entropy, characterized by the absence of any constraint on the freedom of signs to combine with each other. This condition is equivalent to saying that at time *t*_0_, patient and therapist do not share any system of meaning and therefore are in a condition of perfect reciprocal strangeness, i.e., a condition of maximum communicational uncertainty (obviously, this is an idealized condition: even in the first moments of their encounter, patient and therapist possess some shared symbolic background, simply because they are part of a cultural environment).

The authors’ central hypothesis states that, in the first moments of the interaction between patient and therapist, a system of shared meaning suddenly starts to work as the symbolic framework – what the authors call “frame of sense.” This regulates the further communication and, as soon as it is in effect, continues for the remaining interaction. The frame of sense is a phenomenon of *emergence*, consisting of a new organizational structure (a new kind of order) appearing in – and acting on – the dynamics of the system (in the case of psychotherapy, the communication system of therapist and patient). Such a structure consists of a constraint on the associative freedom of the utterances, namely of a reduction of the possibility of combinations of signs – some uttered words will tend to be combined with higher, others with lower probability; some combinations become very improbable.

In order to test their hypothesis, Salvatore et al. (2006/2009) compared the lexical variability characterizing four blocks of five sessions (the first five sessions, two intermediate blocks, and the final sessions). At this aim, they carried out a multidimensional lexical correspondence analyses (MLCA) for each block. Every MLCA was performed on a matrix having utterances in rows and words in columns. Each cell (*ij*) shows whether in the sentence corresponding to row (*i*) the word corresponding to column (*j*) is present or not. Lexical variability was measured in terms of the distribution of the variance associated with the factorial dimensions extracted by the MLCAs: the more dimensions are required in order to explain the inertia the higher the lexical variability. The results showed that lexical variability decreases radically after the first block, remaining constant through the other blocks. Authors interpreted this finding as a marker of a dynamics of emergence of a structure of order consisting in a form of enslaving of the words to a systemic order. This structure of order appears suddenly in the first phase of the life of the system and then remains constant. Moreover, given that it is not the product of an external event (i.e., of an explicit stipulation among patient and therapist), order formation has to be seen as product from within the discursive dynamics, as result of its very functioning. Finally, it can be modeled in terms of reduction of the dimensionality of the phase space, in the case the phase space defined by the factorial dimensions required to describe the lexical variability.

This finding corresponds closely to the pattern formation described by Tschacher et al. (1998) and Tschacher et al. (2007), showing that analogous self-organizing phenomena occur in the psychotherapy system. Synchronization is thus consistently found in datasets of different origin (linguistic, questionnaire, motor behavior).

Two facets lead us to consider particularly promising dynamic models informed by the notion of self-organization for psychotherapy process research. First, the notion of self-organization (i.e., the emergence of macroscopic order as a consequence of the reduction of variability of system components) fits with the clinical and empirical evidence of the multidimensionality of psychotherapy process – namely, of its being a phenomenon entailing a very large number of interacting components at various levels of analysis. Second, the notion of emergence allows to address the transformational nature of clinical process. As already mentioned, clinical exchange is not a mere container – it is a process that changes as a result of its own local output. Patient and therapist form a social cell that generates both formal and informal learning, and in so doing it transforms itself moment by moment. The patient and therapist who conclude a psychotherapy are not the same persons who have initiated it – the dynamics of their encounter, while it has generated some clinical result, has transformed itself, too. This transformation, in our analysis, consists of subsequently emerging patterns of subjective and intersubjective organization, imposing a specific structure of order on the clinical exchange.

## Ways to Grasp Dynamics

In this section we briefly present some procedures and strategies that can be used to perform dynamic analyses of psychotherapy process. We have chosen procedures that we consider suitable for psychotherapy process research both from a conceptual and methodological point of view. Several of our proposals do not require specific technical expertise. Rather, they often suggest a different logic of utilization of well-known procedures of data analysis. The more complicated methods will be anchored to the standard approaches from which they were developed. More sophisticated procedures are available (e.g., cfr. Laurenceau et al., 2007; Collins and Sayer, 2009; Valsiner et al., 2010), but here we wish to show that clinical researchers already have at their hands the analytical resources for performing valid and useful dynamic analyses.

### Modeling non-linear trends

Theory of dynamic systems invites a focus on the global shape of the dynamics of psychotherapy process, on the behavior of the relevant longitudinal parameter(s), rather than on the identification of the absolute magnitudes associated with specific temporal points. The dynamics of the process can be depicted by its trajectory in phase space (see above for a definition). The characteristics of the trajectory (e.g., the presence of extreme values or attractors such as fixed points) allow to describe the dynamics qualitatively as well as quantitatively (Tschacher, 1997).

The study of Barkham et al. (1993) provides an example of this approach. In this study, the main focus of the authors was not on the measurement of variables such as the relevance of the clinical problems brought into psychotherapy. Rather, they focused on modeling the temporal trajectory of the variables under investigation. They tested the fit of a specific model defined by a second order equation. In this way they were able to accept the hypothesis of a *U*-shaped trend in psychotherapy process.

Similarly, Salvatore et al. (2010) conceptualized psychotherapy process using the Two Stage Semiotic Model (TSSM), asserting the *U*-shaped trend of the super-order meanings active in clinically efficacious psychotherapy – i.e., the generalized meanings exerting a regulative function on the patient’s sensemaking, thereby grounding her/his sense of self and world (Salvatore and Valsiner, 2006). Like Barkham et al. (1993), their attention was not on the absolute values of the variables, but on the global shape of the trajectory depicting the course of psychotherapy. In order to subject the *U*-shape hypothesis to empirical scrutiny, the authors analyzed a 15-session good-outcome psychotherapy treatment. For each session an index of the incidence of super-order meaning (Super-order Nodes) was calculated. Then the authors estimated the probability that the observed trend fits a quadratic curve. To this end they calculated the fitted curve’s confidence interval, in order to see if the average absolute value of the residuals lay within it. The mean of the absolute residual value was lower than the confidence interval (at 95%), thus the authors concluded that the Super-ordered Nodes presented *U*-shape course at a significance level of *p* < 0.05. Hence they tested the similarity of the observed curve with the theoretical one (a quadratic curve mapping a *U* trend) by comparing the average differences between the observed and the theoretical values with a 95%-confidence interval.

Recently, Hartmann et al. (2009) presented a regression method with categorical variables assuming non-linear relationships among variables.

Procedures that take non-linear dynamics of phenomena into account are valid as long as stationary trends are analyzed. They are inappropriate, however, in the class of non-linear processes characterized by phenomena of dissipative dynamics and/or emergence of order (see below; Laurenceau et al., 2007; Salvatore et al., 2010).

### Second order trends – mobile difference

In psychotherapy process one usually considers the first order trend of the dependent variable of interest. Nevertheless, clinical experience should lead to take seriously into consideration second order trends of the same variables. While for first order trends one assesses the relation *R* between two states of a variable at two different times, with second order trend (or metatrend) we intend the relation *R′* between two states of *R* at two different times. Acceleration is a classical example of second order trend – it concerns the variation with time of the variation (velocity) of a parameter (movement from a point in space to another).

Clinicians are involved with several facets that should be considered metatrends. The role played by insight in psychodynamic therapy is an example: According to a psychoanalytic standpoint, what is clinically relevant is neither the depth of an insight (of whatever content) nor the frequency of the event conceptualized as insight (first order trends). Rather, what is of clinical interest is the progressive increasing of the magnitude and frequency of the maximum insight reached in each session (a metatrend).

Below we focus on a specific kind of metatrend that we consider clinically relevant: the mobile difference. The clinical meaning of a given state is not provided by its inherent characteristics but by how and how much it is different from the previous state. The relevance of this difference is a common experience of daily life: think of driving a vehicle. After having adjusted to a certain speed, the driver experiences the variations of speed, not the speed itself. In clinical fields, variables such as the therapeutic alliance, defense mechanisms, reflective functioning, and so forth may work and are perceived in terms of their variations, rather than of their absolute levels.

Salvatore et al. (2007) have discussed the empirical relevance such mobile differences may have. They applied DFA (Discourse Flow Analysis) – a novel method aimed at mapping the dynamics of sensemaking sustaining the therapeutic dialog – to the case of Lisa [for further analysis of Lisa’s case, see the special section of *Psychotherapy Research*, volume 19(6), 2008]. DFA depicts each sessions as a set of parameters describing the structural and functional characteristics of verbal exchange between patient and therapist (e.g., level of connectivity among the meanings exchanged between patient and therapist). In addition to the absolute levels of these parameters, the mobile differences (i.e., the variation of the level of the parameter at session *x* + 1 with respect to session *x*) were calculated. Treating the two sets of values (absolute levels and mobile differences) as discriminative variables, the authors were able to distinguish the good-outcome sessions with a 100% rate of success (this was impossible when either absolute levels or mobile differences alone were taken into account as discriminative variables).

Interestingly, a similar result was reported about 25 years ago by Greenberg and Pinsof (1986):

The experience of the Vanderbilt group is particularly illuminating in this regard. Suh et al. (this volume) found that when they examined frequencies of process variables in the first three sessions they found no relationship to outcome, but when they examined the pattern of change in the variables over the first three sessions, process-outcome links began to emerge. Increase in therapist warmth and exploration, and in patient participation over the initial sessions were highly correlated with outcome. (p. 15)

### Pattern analysis

The meaning of events depends on the contexts of co-occurring events. Consequently, what is relevant is not the occurrences in themselves, but the relationships among them. Psychotherapy process is a system: the whole network of relations among elements is different from their additive composition. Elements of the process are like chemical elements: the same elements can produce very different entities just as a consequence of a slight variation of their combination.

The systemic nature of psychotherapy process entails moving the focus of analysis from the individual occurrences of categories of meaning to the patterns of combinations among them (Greenberg, 1994; Salvatore et al., 2009; von Eye et al., 2009). Consider the presence of three categories *a*, *b*, *c*, having occurrences 3, 5, 2 respectively. If one limits the analysis to this frequency distribution one may conclude that categories *a* and *b* are the most present. But this does not necessarily imply that these categories are also the ones endowed with the most relevant causal role. To investigate this role one must map how the categories combine with each other. An identical general distribution like the one above may entail quite different combinatorial patterns, leading to quite different interpretations [e.g., scenario 1: (a-b-b-c), (b-b-c); scenario 2: (a-a), (b-b-b-b-b-c)] (for further considerations of these arguments, see Salvatore and Valsiner, 2010b).

A way to analyze process in term of patterns is provided by multidimensional analysis. Santos et al. (2009) have applied Cluster Analysis (CA) in order to identify patterns of Innovative Moments in a case of good-outcome psychotherapy. Innovative Moments are patients’ narrations introducing novelty to their problematic dominant narratives (Gonçalves et al., 2010). The Innovative Moment Coding System (IMCS, Gonçalves et al., 2010) identifies five categories of Innovative Moments (Action, Reflection, Protest, Re-conceptualization, Performing change), the first three mirroring the emergence of an attitude of rupture with respect to the dominant problematic narrative, the others marking the elaboration of new, less problematic forms of narratives. According to the narrative theory IMCS is grounded in, the clinically positive evolution of the psychotherapy entails a specific pattern of development of the IMs (i.e., the IMs of “rupture” emerge before and pave the way for the “constructive” IMs). Consequently, the analysis of Innovative Moments in psychotherapy cannot be limited to measure the presence of these indicators. Rather, a study of their combination in the different stages of process is also required. CA (hierarchical procedure, based on Ward’s criterion of aggregation) was used to this end. CA is a multidimensional procedure aimed at grouping units of analysis (the rows) according to their similarity on a set of continuous variables (the columns).

Each cluster elaborated by CA was then interpreted as a specific group of sessions characterized by a pattern of IM combinations. In this way, the authors were able to identify three patterns, each of which characterized a stage of the psychotherapy course. This globally supported the hypothesis of the study: Initial sessions (sessions 1–3) were strongly characterized by the prevalence of Action and Reflection; Middle sessions (sessions 4, 5, 7, 8, 9) were characterized by the prevalence of Protest. Some middle and final sessions (sessions 6, 8, 9, 10, 11, 12) were characterized by the combination of constructive IMs (Re-conceptualization and Performing change).

### Sequence analysis

When the psychotherapy course is analyzed using categorical variables that depict temporally discrete states of process, the study of diachronic combinations of states can become relevant. In brief, this approach assumes that a process consists of transitions between non-stationary states of equilibrium. The goals of analysis are to map the modalities and to investigate the conditions and factors involved in the changing of states (for a deeper discussion and more sophisticated models of analysis based on this assumption, see Visser et al., 2010). A basic way of performing this kind of study is sequential analysis (Bakeman and Gottman, 1997; for application of sequential analysis to textual analysis, see Lancia, 2007; to emotion categories, see Reisch et al., 2008). Among the various models of sequential analysis, Markovian analysis is the most feasible and easy to carry out, as well as to interpret. Given a set S of N states, Markovian analysis calculates the probability that state A (predecessor) is followed by state B (successor), both A and B being elements of S. This transition probability is computed as the relative frequency of the successor, i.e., the ratio between the absolute frequency of the successor and the total number of all successors. The result of Markovian analysis quantifies which states follow (i.e., are activated by) a given state, with which probability.

Salvatore et al. (in press) have proposed a strategy of analysis based on the Markovian approach in order to study the relationship between the content of the patient’s narratives and the clinical quality of psychotherapy process. To this end, they classified the sessions of a single case in accordance with an external independent criterion, distinguishing them in good versus non-good sessions. In parallel, they applied a new method of content analysis (DMSC, Dynamic Mapping of the Structures of Content in Clinical Setting), based on six highly general semantic dimensions, each of them articulated in two or three mutually exclusive categories (e.g., the dimension “Reference of the narratives” is articulated in two categories “Self” versus “World”). The transcripts of a patient’s narrative were segmented in Unit of Content (UC; i.e., set of contiguous utterances having an homogeneous semantic content); each UC was coded in accordance with DMSC. Once coded, the UCs were subjected to the following procedure of analysis. Firstly, a multidimensional analysis integrating Multiple Correspondence Analysis (MCA) and CA was performed. By the latter procedure, the UCs were grouped according to their similarities on the DMSC categories; it was analyzed how these categories combined with each other within the patient’s narrative. The clusters of DMSC modalities thus identified were interpreted in terms of *Patterns of content*. Every UC was classified in that Pattern of content that had the strongest association with it.

Secondly, the Probability of Transition PT(*i_t_j*_t + 1_) of each Pattern of content toward each other Pattern of content (including itself) was calculated. The calculations were made separately for each session. PT(*i_t_j_t + 1_*) is the probability that – given a sequence S of a finite set of states M, with states M_i_ *i* = 1, …, *n*, the *i*-th state occurring in the temporal unit *t* is followed by the *j*-th state in the subsequent temporal unit (*t* + 1). PT(*i_t_j_t + 1_*) is calculated as the relative frequency of the sequence:

$$\text{PT }{\left(i_{t}j_{t+1}\right)}_{\text{S}}=k∕p$$

where *k* is the frequency of the occurrences of states *i_t_* − *j_t + 1_* in the sequence of state S and *p* is the frequency of state *i* in the same sequence.

In our case, S is the sequence of Units of Content into which the transcript of the single session was segmented; states *i* and *j* are Patterns of content. For instance, consider that the sequence *i* − *j* is Pattern A – Pattern B. Consider also that Pattern A occurs 10 times in the session under analysis and Pattern B occurs 4 times after that. Therefore, PT(A*_t_*B*_t + 1_*) = *k*/*p* = 4/10 = 0.4.

In the context of our discussion it is remarkable that, while the frequency of Patterns of content in themselves does not discriminate between clinically good and non-good sessions, 8 (out of 25) Probabilities of Transition showed a significant difference between the two classes of sessions – namely, 8 Patterns of content had significantly higher Probabilities of Transition in clinically good sessions than in not-good sessions.

Using an analogous Markovian method, the Adjusted Relative Frequencies (ARF) of transitions between emotion states, Reisch et al. (2008) could distinguish emotion sequences reported by 50 Borderline disorder patients from those of 50 matched healthy persons. The patients showed different attractor emotions as well as generator emotions even after the transition probabilities were adjusted for the different emotion distributions of the two groups.

### Depicting non-stationary phenomena

Self-organization processes introduce a fundamental change in the way of functioning of the system. The fact that a structure of order emerges means that the system modifies its model of behavior in a radical way. Put more formally, the dynamics of a self-organizing system is, by definition, non-stationary because the parameters of the generating equation(s) change with time.

As has been observed in many studies, psychotherapy process is a good candidate for non-stationary dynamics. Therefore, the appropriate method of analysis to be developed should be consistent with this dynamics. Gennaro et al. (2010) provided an example toward this goal. On the base of the *U*-shaped trend of psychotherapy conceptualized by the TSSM (see above), the authors assumed that discursive exchanges between patient and therapist change their fashions of functioning in the two phases of psychotherapy (deconstructive and constructive phase). Assessing this hypothesis, authors split the process in two sets of sessions and separately applied non-parametric analyses of correlations among the DFA indexes. This was done in two different psychotherapies (Lisa and Katja cases). Findings corroborated the hypothesis: in both cases most correlations showed dramatic changes between the two stages – some being significant in the first but not in the second phase, and vice versa; other correlations inverted the direction when changing stages. Only few correlations remained stable.

**Figure 3:**
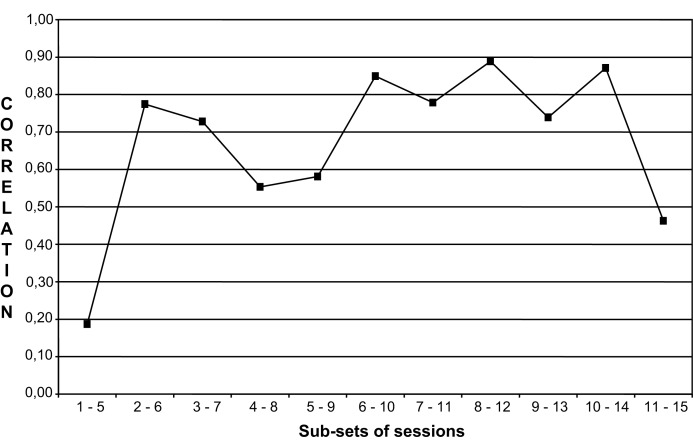
**Correlation between activity and super-order nodes (DFA indexes) within five session subsets from Salvatore et al. (2009)**.

A preliminary study applied DFA to the Lisa case (Salvatore et al., 2007), adopting a further strategy in order to check the emergence of a structure of order in the dynamics of sensemaking. This emergence consists of, and can therefore be marked by, the discrete – rather than incremental – creation of strong associations among the levels and aspects of functioning of the system. This indicates that the system has started to obey a global super-ordered pattern of functioning (i.e., order parameter). In the light of this premise, authors analyzed the relationship between the two main DFA indexes (Activity, AC – an index concerning the capacity of the discourse to produce variability through time – and Super-ordered Nodes, SN – an index marking the incidence of super-ordered meaning active in the discourse) over time. These indexes were chosen because they are assumed to mark two different aspects of system functioning: the former is an index concerning the dynamics of the discursive system; the latter an index concerning the structure and the content of the system. In order to analyze the relationship between Activity and Super-ordered Nodes, authors applied an adapted version of the univariate method of trend analysis proposed by Molenaar and Valsiner (2005/2009). They defined a set of five session blocks with starting session = *n* and ending session = *n* + 4; the blocks were obtained by stepwise varying the cutting point n between *n* = 1 and *n* = 11 (a 5-session range was chosen in order to obtain the highest number of blocks to be compared, yet without compromising the calculation of correlations within each block). The correlation coefficients between AC and SN obtained for each window were then compared. Figure 3 shows the trend of the correlations between the two indexes along the eleven 5-session subsets. Correlation dramatically increased after the first subset of sessions (1–5) – increasing in the following subset (2–6) from *r* = 0.18 to *r* = 0.78. Afterward, the level of correlation stayed fundamentally constant: With the exception of subsets 4–8 and 5–9, the coefficient remained in the range 0.73–0.89 until the last one (even if, due to the small number of cases, the only coefficient statistically significant was the one corresponding to subset 8–12; *r* = 0.89). Authors interpreted this trend as a marker of the initial eruption of emergence of a structure of order, in this case resulting in the coupling of the structural and dynamic dimensions of patient-therapist communication.

A recent development of configural analysis is worth mentioning here – the Configural Frequency Analysis (CFA, von Eye et al., 2009). Broadly speaking, CFA is a useful device for studies where mediation effects are expected, i.e., where an independent variable (predictor) is assumed to have an effect on a dependent variable (criterion) through a third explanatory variable (the mediator). CFA is used to study if and how a predictor has an impact on the criterion. It allows to test the hypothesis of the indirect effect of the predictor, namely that the predictor has an effect on the mediator which in its turn has an effect on the criterion. With respect to other models of configural analysis, CFA has two characteristics that make it suitable for analyzing non-stationary dynamic patterns, i.e., patterns defined by causal relationships among aspects of the process that are not time-invariant. Firstly, CFA addresses mediation effects among categorical variables. Consequently, it is able to recognize specific patterns of mediation, rather than global mediation effects concerning the variables as a whole. Secondly, CFA allows to depict more than one type of mediation effects, each of them concerning a subset of data. Thanks to these two characteristics, CFA can decisively improve the capability of depicting and testing local patterns of interactions among variables – namely patterns that do not concern the whole process and/or all the states of the process, but only a portion of it (i.e., a region of phase space). It is worth noting that CFA can be used irrespective of the role of time. Nevertheless, the model is suitable for encompassing the temporal dimension. In this way it can be used to test if the causal patterns apply only to a phase of the psychotherapy process, in that case highlighting the occurrence of a process of emergence.

## Conclusion

TDS is a quite complicated methodological framework. It entails the need of referring (at least partially) to a formal language, often based on a kind of mathematics the clinical researcher is not familiar with. Moreover, in the light of TDS, most conventional strategies and procedures of data analysis appear obsolete, even lacking validity. Thus, one may ask: why introduce TDS to the clinical field? Should not we defend psychotherapy research against TDS, rather than be pushed to embrace it?

In this paper we have claimed that TDS is a consistent answer to what is obvious for each clinician: the *dynamic nature of the clinical process* – namely, the fact that psychotherapy works *by means* of time, not merely within it. And we have tried to highlight how TDS can be a resource for psychotherapy research. TDS is a rich resource of models and strategies of analysis. Owing to TDS, clinical research can be more consistent with the clinical reality of psychotherapy – thus helping process research to enhance its construct validity.

We are aware that TDS is not only a specific methodology. Rather, it is a paradigmatic way of looking at phenomena that changes the very agenda of the scientific enterprise. It does not only provide new *interpretations* (i.e., what is used for the sake of explanation), but it constructs new *interpretanda* (i.e., what has to be interpreted). To put this in other words, TDS reshapes the scientific enterprise, defining new objects, aims, and standards of knowledge. This is especially true with the concept of emergence, which is more than a descriptive concept: Emergence defines a new class of phenomena in the service of scientific investigation. Moreover, TDS draws a new geography of the boundaries between disciplines. It provides a universal language, allowing the generalization of theories and the exchange among domains of knowledge, in a way that renders obsolete the very distinction between idiographic and nomothetic sciences (Salvatore and Valsiner, 2010a). This is what makes it a promising perspective for the future.

## Conflict of Interest Statement

The authors declare that the research was conducted in the absence of any commercial or financial relationships that could be construed as a potential conflict of interest.
